# Progress towards Mechanism-Based Treatment for Diamond-Blackfan Anemia

**DOI:** 10.1100/2012/184362

**Published:** 2012-04-24

**Authors:** Sara E. Sjögren, Johan Flygare

**Affiliations:** Department of Molecular Medicine and Gene Therapy, 22184 Lund University, Lund, Sweden

## Abstract

Diamond-Blackfan anemia (DBA) is a congenital erythroid hypoplastic anemia, characterized by macrocytic anemia, reticulocytopenia, and severely reduced numbers of erythroid precursors in the bone marrow. For more than fifty years, glucocorticoids have remained the main option for pharmacological treatment of DBA. While continuous glucocorticoid administration increases hemoglobin levels in a majority of DBA patients, it also causes severe side effects. There is therefore a great need for more specific and effective treatments to boost or replace the use of glucocorticoids. Over the years, many alternative therapies have been tried out, but most of them have shown to be ineffective. Here we review previous and current attempts to develop such alternative therapies for DBA. We further discuss how emerging knowledge regarding the pathological mechanism in DBA and the therapeutic mechanism of glucocorticoids treatment may reveal novel drug targets for DBA treatment.

## 1. Diamond-Blackfan Anemia

Diamond-Blackfan anemia (DBA) is a congenital red cell hypoplasia first described by Diamond and Blackfan in 1938 [1, 2]. The disease usually presents within the first year of life as a severe anemia with mild macrocytosis, reticulocytopenia, and a normocellular bone marrow with near absence of erythroid precursors [3, 4]. In addition, patients with DBA typically display elevated erythrocyte adenosine deaminase activity and fetal hemoglobin [3, 5]. The presence of fetal hemoglobin is believed to be an indicator of stress erythropoiesis, while the mechanism behind the elevated adenosine deaminase activity remains elusive. In addition to anemia, around 50% of DBA patients also present physical anomalies, such as craniofacial, urogenital, upper limb, and cardiac malformations [6, 7]. A registry covering many DBA patients in North America (DBA registry (DBAR)) has been established and provides unique data about DBA patients regarding diagnosis, treatment outcomes, and genetics. According to the DBAR, around 31% of all patients are transfusion dependent [6]. Transfusion therapy often has to be combined with iron chelation therapy to avoid iron overload [3, 7]. For a more extensive description of diagnostic criteria and treatment guidelines for DBA we refer to a comprehensive review by Vlachos and Muir and a consensus document by Vlachos et al. [3, 4].

The only curative therapeutic option currently available for DBA patients is hematopoietic stem cell transplantation [8]. However, the procedure is associated with great risks and HLA-matched donors are not always available. During the course of the disease, 17% of all DBA patients enter spontaneous or drug-induced remission, defined as a state of therapy independence for at least six months with physiologically acceptable hemoglobin levels. The mechanism behind remission remains unknown, and around 15% of those who enter remission relapse [6].

## 2. DBA Is Caused by Ribosomal Protein Gene Mutations

While many cases of DBA appear to be sporadic, the disorder can be inherited with incomplete penetrance in an autosomal dominant fashion. The genetic cause of DBA was unknown until the seminal discovery of disruption of the gene encoding ribosomal protein (RP) S19 (RPS19) in a Swedish DBA patient [9]. Since then, eight more ribosomal proteins of the small (S) and large (L) ribosomal subunit have been found mutated in DBA patients, RPS7, RPS10, RPS17, RPS24, RPL5, RPL11, RPS26, and RPL35A [10–14]. However, *RPS19* remains the most commonly mutated gene, responsible for 25% of DBA cases [6, 9]. Ribosomal protein mutations in DBA patients are only found in one allele, with intact RP expression from the other allele [7]. Despite extensive sequencing efforts, only 50% of DBA patients have known mutations in ribosomal protein genes, leaving the other half of patients with unknown genetic cause of the disease [15]. The fact that all known DBA genes are RP genes suggests that DBA is a disorder of faulty ribosome biogenesis.

## 3. DBA Gene Therapy

Since many cases of DBA are caused by a haploid deficiency of an RP, enhanced expression of the specific RP gene using gene therapy has the potential to cure DBA. Hamaguchi et al. have shown that RPS19-deficient CD34^+^ bone marrow cells from DBA patients transduced with lenti- and oncoretroviral vectors containing *RPS19* have increased proliferative capacity compared to control DBA cells [16, 17]. Moreover, CD34^+^ cells from an RPS19-deficient DBA patient transduced with *RPS19* had a proliferative advantage over nontransduced RPS19-deficient cells after transplantation into immunodeficient mice [18]. These results demonstrate that if RP gene transfer can be performed in a safe and efficient fashion, gene therapy has great potential to cure DBA patients.

## 4. Ribosomal Protein Deficiency and p53 Activation Cause Bone Marrow Failure and Anemia

Development of better pharmacological treatments for DBA would be greatly facilitated if the underlying pathogenic mechanisms of the disease were completely understood. While several early studies suggested that DBA was caused by immune reactions or other extrinsic factors, we now know that the pathogenic mechanism is linked to intrinsic ribosomal protein deficiency [19]. Further evidence supporting this conclusion comes from studies by us and others showing that overexpression of *RPS19* rescues the DBA phenotype in RPS19-deficient DBA patient cells. Furthermore, RNA interference-induced knockdown of RPS19 in normal cells induces a DBA phenotype, including apoptosis and reduced proliferation of erythroid precursors similar to that observed in DBA patient cells [16–21]. We and others have shown that RP-deficient cells progressing from erythroid colony forming unit (CFU-E) to the erythropoietin- (Epo-) dependent terminal erythroid differentiation are displaying apoptosis and cell cycle arrest [22, 23]. The arrested proliferation is associated with upregulation of p53 target genes, suggesting that DBA in part is caused by *p53* activation [24]. Accordingly, elevated levels of p53 have been demonstrated in other ribosomal disorders such as 5q myelodysplastic syndrome (5q^−^) and Treacher-Collins syndrome (TCS) [25–27]. Furthermore, partial or full knockout of *p53* in animal models lacking the genes *Rps14* in 5q^−^ and *Tcof1* in TCS dramatically ameliorated the phenotype of both disease models [25, 27]. This is in accordance with previous studies showing that cell cycle arrest in RPS19- and RPS14-deficient erythroid precursors is caused by up-regulation of *p53* [26]. A zebrafish model of *RPS19*-deficient DBA displays defects in definitive erythropoiesis and congenital anomalies. The congenital anomalies could be rescued by knockout of *p53*, while the erythropoiesis remained defective [28]. An other recently published study has found that erythroid defects of an RPS29-deficient zebrafish can nearly completely be reversed by introducing a mutation that abolishes DNA-binding capacity of p53 [29]. It is previously known that cells of the erythroid lineage are very sensitive to *p53* up-regulation and that *p53* activation induces premature maturation [26]. Recently, Jaako et al. have confirmed that *p53* indeed plays a role in DBA pathogenesis, showing that anemia and bone marrow failure in Rps19-deficient mice are almost completely rescued in a *p53*-null background [30]. Hence, *p53* activation may at least partially explain why the erythroid lineage is specifically affected in DBA.

While it is evident that *p53* activation plays an important role in DBA, it is not yet understood how RP deficiency induces *p53* activation or which of the *p53* target genes are responsible for the phenotype. It has been hypothesized that the mechanism leading to *p53* activation in RPS19-deficient cells is similar to that of actinomycin D-induced ribosomal stress. Actinomycin D inhibits RNA polymerase I-dependent rRNA transcription, which causes free RPs to leak out from the nucleolus, such as RPL5 and RPL11. By their release, they are able to specifically bind and inhibit MDM2/HDM2, preventing it from promoting ubiquitination and degradation of p53, leading to induced expression of *p53* target genes, cell cycle arrest, and apoptosis [31, 32]. Thus, ribosomal proteins are directly involved in cell cycle control by inducing or stabilizing the expression of *p53* [31–33]. Although several researchers have put this theory forward, it has never been confirmed to be a mechanism in DBA [31, 34, 35]. It has however been shown that a haploinsufficient level of *RPS19* arrests ribosomal biogenesis, disrupts ribosomal RNA (rRNA) maturation, and causes up-regulation of genes downstream of *p53* [24, 26, 28, 36]. Cells from RPS19-deficient DBA patients thus have all the hallmarks of “ribosomal stress.” Conclusively, these findings emphasize the important role of elevated *p53* levels in DBA pathogenesis and raise the question if drugs could be developed that specifically prevent *p53* up-regulation in response to RP deficiency.

## 5. Increased Knowledge of the Therapeutic Response to Glucocorticoids Could Lead to New Treatments

Glucocorticoids (GC), today mostly in the form of prednisone, have remained the drug of choice for DBA patients since their first reported use in the 1950s [4, 37, 38]. While about 80% of all DBA patients initially respond to GC treatment, one in two eventually discontinue GC treatment due to loss of response or severe side effects [6]. Side effects associated with GC treatment in children with DBA are common and include growth retardation, bone fractures, severe infections, cataracts, hypertension, and diabetes mellitus [6, 7]. Glucocorticoids are therefore rarely used during the first year of life [4, 39, 40]. Although prednisone remains a good option for many patients, enhanced understanding of its therapeutic mechanism in DBA could lead to new and better tolerated DBA drugs.

The mechanism underlying the therapeutic effect of GC is not fully understood, and it remains elusive if the effect is disease-specific or merely the result of a direct stimulatory effect on self-renewal of early erythroid precursor cells [41–43]. The latter is supported by the physiological role of the endogenous GC cortisol in promoting self-renewal of erythroid precursors and rapid normalization of severe anemia [44–46]. Interestingly the GC-induced response to stress erythropoiesis is accelerated in *p53* knock-out mice [47], which suggests a reciprocal role of the GC receptor and p53 in regulation of erythropoiesis, maintaining the balance between self-renewal and differentiation. It is therefore possible that the therapeutic effect of prednisone in part is disease-specific and involves mechanisms that counteract *p53* activation in erythroid precursors.

## 6. Clinically Tested Alternative DBA Therapies

Over the years there have been several reports of patients entering remission during the treatment with a particular drug. In the search for new and better DBA drugs, several compounds have been tested based on previously proposed mechanisms. While few of these drugs proved useful in more than occasional cases, these attempts contributed to valuable insights into DBA pathogenesis.  Table 1 lists and summarizes the clinical outcome of several different therapies tried in DBA patients.

### 6.1. EPO

Erythropoietin (Epo) is endogenously produced by the kidney in response to hypoxia and promotes proliferation, survival, and differentiation of CFU-E precursor cells to orthochromatic erythroblasts, while neither earlier precursors, such as burst-forming unit erythrocyte (BFU-E), nor differentiation beyond orthochromatic erythroblasts depends on Epo [63–65]. Although DBA patients display high concentrations of circulating Epo as the system is trying to increase red cell production, they are typically unresponsive to Epo stimulation, likely due to lack of erythroid precursors expressing the Epo receptor [1, 52, 53, 66]. The fact that DBA patients do not respond to Epo suggests that new treatment strategies should aim to increase the number of Epo-responsive erythroblasts in the circulation. This could potentially be accomplished either by counteracting the DBA-specific mechanism that prevents formation of Epo-responsive erythroblasts or by stimulating self-renewal of early erythroid precursors through mechanisms related to those promoting production of Epo-responsive erythroblasts during stress erythropoiesis.

### 6.2. Interleukin 3

Interleukin 3 (IL-3) is known to stimulate erythropoiesis at an earlier stage than Epo and was therefore considered a possible DBA candidate drug. Interleukin-3 promotes cell cycle progression and survival through the JAK/STAT, RAS/MAP, and PI3K signaling pathways [67] and sustains survival of erythroid cells by upregulating the antiapoptotic factor Bcl-2 via the PKC pathway [68]. Clinical trials evaluating IL-3 as a DBA therapy demonstrated good response in some patients [54–57]. While it was relatively well tolerated in the majority of patients, one study reported cases of deep venous thrombosis [56]. A recent consensus conference concluded that the number of patients responding to IL-3 treatment remains too small to consider it a satisfactory option for first-line treatment [4].

### 6.3. Other Compounds

One case of a DBA patient experiencing remission from anemia when breast-feeding led to a study using the prolactin-inducing agent metoclopramide to treat DBA. Positive effects were seen in three of nine patients [60]. However, a later study could not confirm the positive effects of metoclopramide in DBA patients [61].

In another case report, a DBA patient entered remission after receiving valporic acid for seizure therapy [62]. Valporic acid is a histone deacetylase inhibitor, which is a group of compounds known to promote survival and proliferation by repressing PUMA and Bax-mediated apoptosis in neuronal cells [69]. However, there is no evidence for an antiapoptotic effect on DBA erythroid precursors, and clinical trials with more patients have not been carried out.

Cyclosporine A is an immune suppressant, which also has been investigated as a possible DBA drug based on the erroneous hypothesis that DBA is an immune disorder [48, 70]. Nevertheless, there are several reports of DBA patients benefitting from cyclosporine A treatment that might be explained by intrinsic effects on erythropoiesis by this drug, separate from its immunomodulatory actions [48, 49]. Further studies are required to determine if metoclopramide, cyclosporine A, or valproic acid are able to modulate the DBA disease phenotype.

When culturing cells *in vitro*, the c-Kit ligand stem cell factor (SCF) has proven important for proliferation of erythroid progenitors and can enhance their cell cycle progression. The clinical use of SCF might however be limited due to its effect on mast cell degranulation, which can cause allergic reactions [71].

## 7. Lenalidomide and Leucine Are Currently Evaluated in Clinical Trials as DBA Treatments

As of December 2011, two clinical trials are recruiting patients to evaluate the therapeutic effect of the potential DBA drugs lenalidomide and leucine. Lenalidomide is a thalidomide derivative with anti-inflammatory and anti-tumor properties that is currently used to treat 5q^−^ [72]. 5q^−^ is a myelodysplastic syndrome subtype characterized by defective erythroid differentiation due to loss of function of *RPS14*, which links the molecular pathophysiology of 5q^−^ to DBA [73]. Since ribosomal protein deficiency and *p53* up-regulation contribute to anemia in both 5q^−^ and DBA, DBA patients might respond to lenalidomide in a similar in fashion as 5q^−^ patients [22, 26]. In addition to potential disease-specific effects on erythropoiesis, lenalidomide has been shown to increase the generation of normal human CFU-E precursors from CD34^+^ cells [41].

The second compound currently in clinical trial is the branched amino acid leucine. Leucine and its metabolite beta-hydroxy-beta-methylbutyrate stimulate initiation of protein translation through activation of the mTOR complex [74–76]. The hypothesis behind this study is that leucine will compensate for the decreased rate of protein translation caused by RP deficiency seen in DBA patients [77]. Supported by a pilot study, leucine administration indeed coincided with remission in one DBA patient [78]. At the 2011 ASH meeting, Jaako et al. presented data indicating that leucine administration partially rescues the erythrocyte numbers in RPS19-deficient mice [79].

## 8. Glucocorticoid Receptor Agonist Replacement with Fewer Side Effects

Like prednisone, there are several steroid and nonsteroid compounds that are potent GC receptor agonists. Since some of these ligands only partially activate the receptor, it may be possible to identify GC receptor agonists that at certain doses retain the therapeutic effect in DBA patients without inducing any side effects. While no such drugs are currently available, there have been attempts to test safer GC receptor agonists such as deflazacort (DFZ) in DBA. Deflazacort is a bulky derivative of prednisolone with reportedly milder side effects on growth of long bone in children at similar dosages. Currently, DFZ is used as an alternative to prednisone in children with Duchenne's muscular dystrophy and has successfully been used in at least one DBA patient [80]. It remains to be tested if DFZ results in less severe side effects compared to prednisone also in children with DBA.

## 9. Reduction of Glucocorticoid Dose by Combination Therapies

Recently, Narla et al. demonstrated that GC-induced proliferation of erythroid precursors was augmented when lenalidomide was combined with dexamethasone. Interestingly, dexamethasone stimulated proliferation of normal BFU-Es while lenalidomide mainly enhanced proliferation of CFU-Es [41]. The two compounds further upregulated a different set of genes, indicating that they act through two distinct mechanisms. The authors propose that a combinatory therapy could be an option for patients with poor response to normal GC treatment.

Another group of drugs that could possibly be used to enhance the effect of GC are drugs that promote activation of hypoxia-inducible factor 1*α*  (HIF1*α*), so-called prolyl hydroxylase inhibitors. We have previously demonstrated in normal BFU-E's that genes regulated by GC also contain binding sites for HIF1*α*, indicating that these transcription factors might enhance each other's functions. Indeed, co-treatment with prolyl hydroxylase inhibitors that inhibit HIF1*α*  degradation increased the expression of GC-induced genes and boosted the proliferative capacity of GC treated BFU-E cells *in vitro *[42]. It remains to be tested if prolyl hydroxylase inhibitors or other drugs that enhance HIF1*α*  activation rescue the DBA phenotype in hematopoietic cells from DBA patients or in any of the available DBA mouse models [36]. With regard to HIF1*α*, it is interesting to note that there are several reports of iron chelation therapy in patients with myelodysplastic syndrome and DBA, leading to a rapid increase in red cell production [51, 81]. While this therapeutic response likely is linked to decreased iron toxicity on bone marrow erythropoiesis, iron chelators are also potent HIF1*α*  activators. It is therefore possible that the observed increase in red cell production is in part attributed to HIF1*α*  activation in BFU-E precursors [42, 82, 83]. If iron chelation has this additional effect related to HIF1*α*  activation in DBA patients, the response to GC could be more pronounced if combined with iron chelators.

## 10. Future Directions

**Figure 1:**
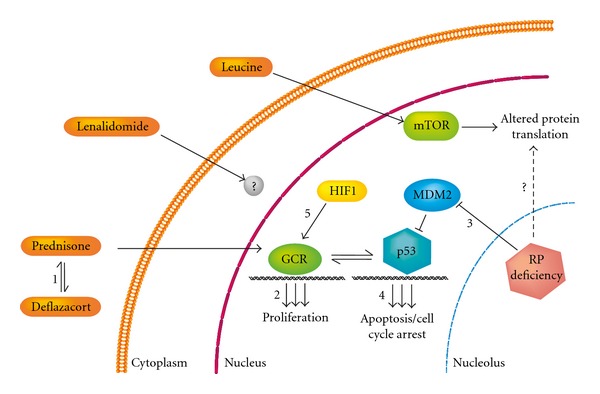
Pathways involved in DBA pathogenesis and possible targets for new drugs. (1) Replacement of prednisone with a GC receptor agonist with potentially less side effects, for example, deflazacort. (2) Identification of genes involved in the therapeutic response to prednisone and subsequent identification of compounds that specifically induce or activate these genes. (3) Identification of genes and pathways involved in the mechanism by which ribosomal stress induces a *p53* response. (4) Identification of genes that act downstream of p53 to induce the DBA phenotype. (5) Enhance the effect of GC in combination with other compounds, for example, stabilizers of HIF1*α*.

In order to develop new drugs for DBA, it is crucial to gain further insight into the regulatory pathways and key genes involved in erythropoiesis. Diamond-Blackfan anemia is a complex disorder, and the defective ribosomal biogenesis affects different pathways within the early erythroid precursors. Despite being the first treatment option, GC are associated with many side effects, and some of them are severe. For this reason, many alternative pharmacological compounds have been tried over the years. As presented in this paper, few of them have proved efficient in DBA patients. Presently several research groups are putting great effort into fully understanding DBA pathology. The identification of more DBA genes and detailed understanding of molecular pathways causing anemia in DBA will likely provide several possible DBA drug targets. In Figure 1, we have summarized the current but incomplete understanding of DBA pathogenesis and highlighted five areas where potential target genes for new therapies may be identified.

Ideally, some of the pathways involved in DBA pathogenesis or the therapeutic response to prednisone contain a few of the estimated 2000–3000 genes in the genome that are considered “druggable” [84]. For the purpose of developing new DBA drugs, it is therefore crucial to perform studies aimed at mapping all pathways involved in DBA pathogenesis. When the secrets of DBA pathogenesis are uncovered, the search for drugs targeting specific DBA-modifying genes can truly begin.

## Figures and Tables

**Table 1:** Compounds clinically tested as new DBA treatments and their respective outcome.

Therapy	Patients (*n*)	Positive responders	Comments	Ref.
Cyclosporine A	19	22–50%	Good long-term response for some, but with side effects	[48–50]
Deferasirox	1	100%	Similar effect observed in MDS patients	[51]
Erythropoietin	10	0%	No response	[52, 53]
Interleukin 3	92	12–22%	Only partial response in some patients. Some severe side effects	[54–57]
IV IgG	2	0%	Brief or no response	[58, 59]
Metoclopramide	42	6–33%	Two studies with contradicting results	[60, 61]
Valproic acid	1	100%	Patient in sustained remission	[62]
